# Haimufang decoction, a Chinese medicine formula for lung cancer, arrests cell cycle, stimulates apoptosis in NCI-H1975 cells, and induces M1 polarization in RAW 264.7 macrophage cells

**DOI:** 10.1186/s12906-020-03031-1

**Published:** 2020-08-05

**Authors:** Wei-Ping Ma, Shu-Man Hu, Yan-Lai Xu, Hai-Hua Li, Xiao-Qing Ma, Bao-Hong Wei, Fu-Yu Li, Hua-Shi Guan, Guang-Li Yu, Ming Liu, Hong-Bing Liu

**Affiliations:** 1 Key Laboratory of Marine Drugs, Chinese Ministry of Education, School of Medicine and Pharmacy, Ocean University of China, Qingdao, 266003 China; 2 Marine Biomedical Research Institute of Qingdao, Qingdao, 266071 China; 3 Naval Secret Service Nursing Center of Qingdao, Qingdao, 266071 P. R. China; 4 Laboratory for Marine Drugs and Bioproducts, Pilot National Laboratory for Marine Science and Technology, Qingdao, 266237 China

**Keywords:** Haimufang decoction (HMF), Cell cycle, Apoptosis, Immunomodulation, M1 phenotype polarization

## Abstract

**Background:**

Lung cancer has the highest morbidity and mortality in the world and novel treatment strategies are still needed. Haimufang decoction (HMF) is a patented clinical prescription of traditional Chinese medicine for lung cancer treatment. HMF is composed of four herbs and has been applied clinically in advanced cancer patients. However, its therapeutic mechanisms are still unclear. This study aims to elucidate the possible mechanisms of HMF for the treatment of lung cancer.

**Methods:**

3-(4,5-dimethyl-2-thiazolyl)-2,5-diphenyl-2-H-tetrazolium bromide assay was applied for evaluating the proliferative effect of HMF in lung cancer cells and monocyte macrophage RAW264.7 cells. Flow cytometer was used to detect the effects of HMF on cell cycle and apoptosis, and western blotting was employed to explore the potential apoptotic mechanisms of HMF on lung cancer cells. For immunomodulatory effect, co-culture system was used to detect the activation of macrophage RAW264.7 cells when treated with HMF, and neutral red assay was used to measure the effect of HMF on the phagocytosis of the activated macrophages. Enzyme linked immunosorbent assay, flow cytometer, and immunofluorescence staining method were employed for the investigation on the underlying mechanisms of the immunomodulatory effect on RAW264.7 induced by HMF.

**Results:**

HMF inhibited the proliferation, induced S phase cell cycle arrest, and stimulated apoptosis in lung cancer NCI-H1975 cells, while had negligible cytotoxicity on macrophage RAW264.7 cells. Moreover, HMF could activate macrophage RAW264.7 cells and promote the inhibition activity of RAW264.7 cells against lung cancer cells. And also, HMF activated macrophages and increased their phagocytic activity in a concentration-dependent manner. HMF increased the expression of macrophage activation marker CD40, the level of nitric oxide, the generation of intracellular reactive oxygen species, as well as M1 macrophages cytokines including tumor necrosis factor-*α*, interleukin-1*β*, interleukin 12 p70, and interleukin 6. Further investigation showed that HMF induced M1 but not M2 phenotype polarization in RAW264.7 cells.

**Conclusions:**

HMF can mainly exert anticancer activity via (1) cytotoxicity to human lung cancer cells by proliferation inhibition, cell cycle arrest, and apoptosis induction; and also via (2) immunomodulation via macrophage cells activation and M1 phenotype polarization induction.

**Graphical Abstract:**

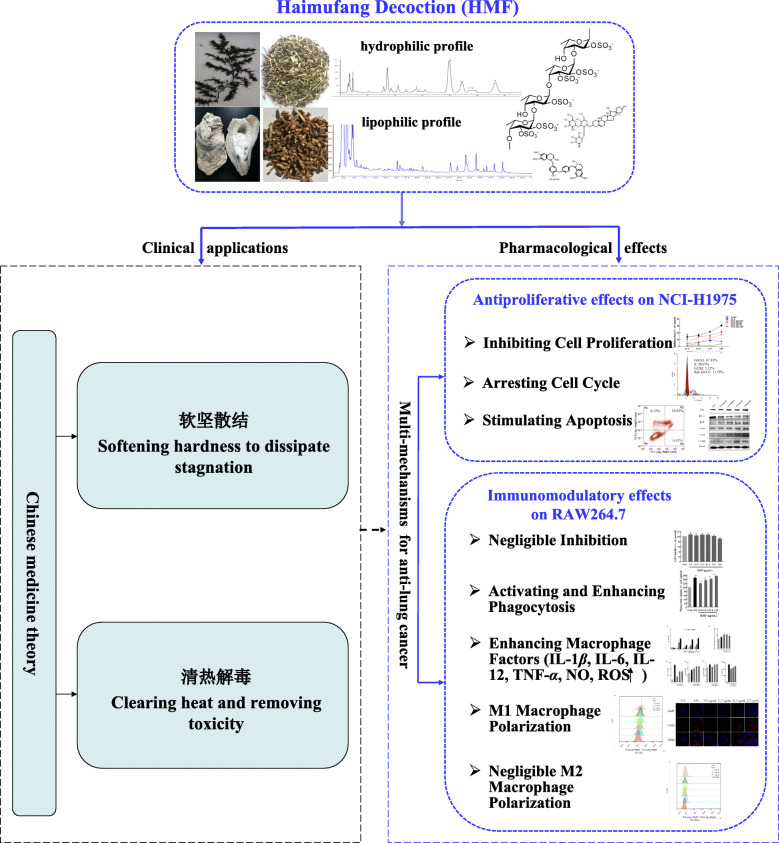

## Highlights

- HMF inhibits proliferation, arrests cell cycle, induces apoptosis in cancer cells.
- HMF exerts immunomodulatory effect of macrophages.
- HMF activates and induces M1 phenotypic polarization in RAW264.7 cells.

## Background

Lung cancer is the highest morbidity disease and still the leading cause of cancer mortality worldwide [1]. Despite major advances in the clinical treatments, the 5-year survival rate of non-small cell lung cancer (NSCLC) is still as low as 10% [2], and the main conventional chemotherapy drugs are still dismal due to serious side effects and drug resistance [3]. Therefore, effective and alternate treatment strategies are urgently needed.

Traditional Chinese medicine (TCM), an important component of the world’s national medicine, shows good therapeutic effects in various cancers with low toxicity [4]. The mechanisms underlying the anticancer action of TCM mainly include: cytotoxicity, immunomodulation, tumor metastasis suppression, or gut microbiota modulation [5]. TCM has unique advantages in treating cancers because of its comprehensive pharmacological effects of multi-channel, multi-levels, and multi-targets. For example, Chinese medicine formula Tian Xian Liquid, inhibits tumor growth effectively [6] and also enhances immunity by promoting macrophages proliferation [7].

Haimufang decoction (HMF) is a patented clinical prescription for advanced lung cancer. We previously found that HMF could significantly inhibit the growth of transplanted lung cancer in mice, prolong the survival of tumor-bearing mice, and ameliorate the life quality of tumor-bearing mice with good safety [8]. It contains two marine original medicinal slices (Sargassum (frond of *Sargassum fusiforme* (Harv.) Setch), “Hai-zao” in Chinese) and Ostreae Concha (shell of *Ostrea gigas* Thunberg, “Mu-li-ke” in Chinese), and two terrestrial original medicinal slices (Menispermi Rhizome (rhizome of *Menispermum dauricum* DC.), “Bei-dou-gen” in Chinese) and Solani Nigri Herba (aerial part of *Solanum nigrum* L., “Long-kui” in Chinese). Brown algae *Sargassum fusiforme* which dosage is twice than others, contains abundant polysaccharides such as fucoidan, algin, and so on. There are some evidences that fucoidan exerts anticancer activity through immune regulation [9, 10]. In addition to polysaccharides, HMF contains quite a few of alkaloids, including dibenzylisoquinoline alkaloids dauricine and daurisoline which derived from *Mertispermum dauricum*, and steroid alkaloids solasonine and solamargine from *Solanum nigrum.* Although HMF is effective in clinical use as well as in animal models [8], the possible mechanisms of HMF underlying the treatment of lung cancer are still unclear.

In this study, we first investigated the effect of HMF on lung cancer cells by evaluating the NCI-H1975 cells proliferation, cell cycle distribution, and apoptosis after HMF treatment. Furthermore, we also detected the immunomodulatory effect of HMF on macrophage cells by detecting the activation and polarization of RAW264.7 cells, and explored the related mechanisms.

## Methods

### Plant materials

All the medicinal slices, Sargassum (frond of *Sargassum fusiforme* (Harv.) Setch, *Batch No.* 170601, produced in Zhejiang province, China)*,* Ostreae Concha (shell of *Ostrea gigas* Thunberg, *Batch No.* 170601, produced in Shandong province, China), Menispermi Rhizome (rhizome of *Menispermum dauricum* DC., *Batch No.* 161101, produced in Shandong province, China), and Solani Nigri Herba (aerial part of *Solanum nigrum* L*.*, *Batch No.* 170301, produced in Shandong province, China) were provided by Pharmacy of the Affiliated Hospital of Qingdao University and were authenticated by professor Feng-Qin Zhou (from Shandong University of Traditional Chinese Medicine) according to the Pharmacopoeia of the People’s Republic of China identification key (2015, Volume 1). Voucher specimens’ numbers of Sargassum, Ostreae Concha, Menispermi Rhizome and Solani Nigri Herba were YP-Z-HZ.12, YP-D-ML.28, YP-TZ-BDG.7 and YP-TZ-LK.3, respectively. Voucher specimens of these medicinal slices were deposited at the Key Laboratory of Marine Drugs, the Ministry of Education of China, Ocean University of China, Qingdao, China.

### Preparation of HMF extract

The preparation of HMF was decocted for traditional method and provided by Marine Biomedical Research Institute of Qingdao (Qingdao, Shandong, China). In brief, the medicinal slices of Sargassum (frond of *Sargassum fusiforme* (Harv.) Setch), Ostreae Concha (shell of *Ostrea gigas* Thunberg), Menispermi Rhizome (rhizome of *Menispermum dauricum* DC*.*) and Solani Nigri Herba (aerial part of *Solanum nigrum* L.) were mixed as a proportion of 6:3:3:2, and the total dry weight was 5 kg. The mixture was decocted in 50 L distilled water (100 °C) for 1 h and repeated once. The combined water extracts were immediately filtered through 200 mesh, centrifuged (3000 rpm/min, 10 min), and concentrated followed by freeze dried to obtain powder for use. 1 g dried powder contains 7.81 g total original herbs.

### Chemicals and reagents

Roswell Park Memorial Institute (RPMI)-1640 medium, Dulbecco’s modified Eagle’s medium (DMEM), Ham’s F-12 K (F-12 k) medium were purchased from Gino Biomedicine Technology Co., Ltd. (Hangzhou, Zhejiang, China). Fetal bovine serum (FBS) was obtained from Genetimes Technology Inc. (Shanghai, China). Cell cycle and apoptosis analysis kit, total nitric oxide (NO) assay kit, reactive oxygen species (ROS) assay kit, 3-(4,5-dimethylthiazol-2-yl)-2,5-diphenyltetrazolium bromide (MTT) were purchased from Beyotime Biotechnology Co., Ltd. (Shanghai, China). Lipopolysaccharides (LPS), bovine serum albumin (BSA), 4′,6-diamidino-2-phenylindole (DAPI), and phosphate buffer saline (PBS) were obtained Beijing Solarbio Science & Technology Co., Ltd. (Beijing, China). The recombinant mouse interleukin (IL) 4 protein was obtained from Beijing Sino Biological Inc. (Beijing, China). Mouse tumor necrosis factor-*α* (TNF-*α*) elisa kit, mouse interleukin-1*β* (IL-1*β*) elisa kit and mouse interleukin 12 p70 (IL-12 p70) elisa kit were purchased from Dakewe Biotech Co., Ltd. (Shenzhen, Guangdong, China). Mouse interleukin 6 (IL-6) elisa kit, allophycocyanin (APC)-conjugated anti-mouse cluster of differentiation (CD) 86 antibody, and fluorescein isothiocyanate (FITC)-conjugated anti-mouse F4/80 antibody were purchased from Multi science (Lianke) Biotech, Co., Lit. (Hangzhou, Zhejiang, China). BCL-2 associated X protein (BAX) monoclonal antibody, P53 monoclonal antibody, and goat anti rabbit immunoglobulin G (IgG)-horseradish peroxidase (HRP) secondary antibody were purchased from Hangzhou Huaan Biotechnology Co., Ltd. (Hangzhou, Zhenjiang, China). Antibodies against B-cell lymphoma-2 (BCL-2), cleaved-cysteinyl aspartate specific proteinase-3 (C-caspase 3), cleaved-cysteinyl aspartate specific proteinase-9 (C-caspase 9), cleaved-poly-adenosine diphosphate-ribose polymerase (C-PARP), were purchased from Cell Signaling Technology (Boston, MA, USA). APC-conjugated anti-mouse CD206 monoclonal antibody and phycoerythrin (PE)-conjugated anti-mouse CD40 monoclonal antibody were purchased from Elabscience Biotechnology Co., Ltd. (Wuhan, Hubei, China).

### HPLC fingerprints of HMF for quality control

For analyzing the hydrophilic profile of HMF (identification of monosaccharides): an Agilent series 1260 Infinity HPLC instrument equipped with diode array detector was performed. The chromatography conditions were as follows: COSMOSIL 5 C18-PAQ column (250 × 4.6 mm, 5 μm); mobile phase, 100 mM phosphate buffer and acetonitrile at a ratio of 82:18 (v/v, %); flow rate, 1 mL/min; column temperature, 30 °C; injection volume, 10 μL; detector wavelength, 254 nm.

For analyzing the lipophilic profile of HMF (identification of alkaloids): UltiMate 3000 HPLC equipped with an Alltech 2000ES Evaporative Light Scattering Detector (ELSD, Alltech, USA), SinoPack C18 column (250 × 4.6 mm, 5 μm); flow rate, 1 mL/min; column temperature, 35 °C; injection volume, 10 μL; the gradient elution procedure of mobile phase A (acetonitrile) and mobile phase B (0.01% triethlamine water) was as follows: 0–12 min, 25–36% A; 12–16 min, 36–39% A; 16–36 min, 39–43% A; 36–45 min, 43–100% A; 45–52 min, 100% A; 52–53 min, 100–25% A; 53–60 min, 25% A. The ELSD was set as follows: drift tube temperature was set at 105 °C, nitrogen gas as carrier gas and the flow rate was set at 3.2 mL/min.

### Directly antiproliferative effect of HMF on lung cancer cells

#### Cell lines and cell culture

Mouse lung cancer cell line Lewis (LLC), human non-small cell lung cancer cell lines NCI-H1975, NCI-H1299, NCI-H460, NCI-H292, and A549 were provided by Marine Biomedical Research Institute of Qingdao (Qingdao, Shandong, China). Mouse monocyte macrophage (RAW264.7) cells were purchased from Shanghai Institute of Biochemistry and Cell Biology (Shanghai, China). A549 cell line was cultured in F-12 K medium with 10% (v/v) FBS and 1% penicillin/streptomycin. Others were cultured in RPMI-1640 medium with 10% FBS and 1% penicillin/streptomycin. RAW264.7 cells were cultured in DMEM supplemented with 10% FBS, 100 U/mL penicillin and 100 mg/mL streptomycin. All the cells were cultured at 37 °C with 5% CO_2_ in a humidified incubator.

#### Cell cycle analysis assay

NCI-H1975 cells were seeded in six-well plates (2 × 10^5^ cells/well), and treated with RPMI-1640 medium with different concentrations (31.3–250 μg/mL) of HMF for 48 h. The control group was treated with RPMI-1640 medium with 10% FBS. Cells were collected, washed, and fixed with 70% cold ethanol overnight at 4 °C. Then cells were treated with 500 μL propidium iodid (PI, 50 μg/mL) solution containing 100 μL ribonuclease (100 μg/mL) for 30 min at 4 °C in the dark. The cell cycle distribution was detected by flow cytometry (Beckman Coulter MoFlo XDP, Fullerton, CA, USA), and the data was analyzed with ModFit LT software (Verity Software House. Inc., Topsham, ME, USA).

#### Annexin V-FITC/PI analysis assay

NCI-H1975 cells were seeded in six-well plates (3 × 10^5^ cells/well), and treated with RPMI-1640 medium with different concentrations (31.3–250 μg/mL) of HMF for 48 h. After HMF treatment, apoptosis was detected using an Annexin V-FITC apoptosis detection kit, according to the manufactures’ protocol. Briefly, cells were treated with different concentrations of HMF, harvested and washed with ice-cold PBS, and then resuspended in 100 μL binding buffer containing 5 μL Annexin V-FITC and 5 μL PI. After incubation for 15 min in the dark at room temperature, cells were analyzed on an Aria FACS flow cytometry system (Beckman Coulter MoFlo XDP, Fullerton, CA, USA).

#### Western blot

NCI-H1975 cells were seeded in six-well plates (5 × 10^5^ cells/well), and treated with RPMI-1640 medium with different concentrations of HMF (31.3–250 μg/mL) for 48 h. Proteins were collected from the lysed cells on ice, separated on 10% SDS-polyacrylamide gels, and transferred to nitrocellulose membranes. The membrane was blocked and incubated with primary antibodies overnight at 4 °C, and then incubated with horseradish peroxidase or alkaline phosphatase-coupled secondary antibody at room temperature. *β*-actin was used as the internal loading control. Specific proteins were detected with enhanced chemiluminescense by FluorChem E (Protein Simple, San Jose, CA, USA).

### Immunomodulatory effect of HMF on RAW264.7 cells

#### Antiproliferation activity of activated RAW264.7 cells

RAW 264.7 cells were plated at a density of 4 × 10^4^ cells per well in 96-well plates. After cell attachment, the medium was replaced with LPS (1 μg/mL) or various concentrations of HMF (15.6–125 μg/mL). After 24 h, each well was added with different lung cancer cells lines (macrophages: tumor cells, i.e., effector: target cells = 20:1). Antiproliferation activity of activated RAW264.7 against the targeted cells was determined by MTT assays.

#### Phagocytic activity of RAW 264.7 cells

Effect of HMF on the phagocytosis of RAW 264.7 cells was measured by neutral red uptake method [11]. In brief, RAW 264.7 cells (5000 cells/well) were seeded into 96-well plates, and incubated for 4 h in 5% CO_2_ atmosphere at 37 °C. Then the medium was replaced with LPS (1 μg/mL) or various concentrations of HMF (15.6–125 μg/mL). After incubation at 37 °C for another 24 h, the medium was discarded and replaced with 0.1% neutral red physiological saline (100 μL/well). After further incubation for 30 min, the plates were washed with PBS for 3 times. Pyrolysis liquid (acetic acid: ethanol = 1:1, 150 μL/well) was added into each well. The absorbance at 570 nm was measured and the phagocytosis was expressed as OD values.

### Measurement of intracellular NO and ROS

RAW264.7 cells were treated with LPS (1 μg/mL) or different concentrations of HMF. After incubation for 24 h, the incubated RAW264.7 cells were washed 3 times with PBS and then added with 10 μM fluorescent probe 2′,7′-dichlorodihydrofluorescein diacetate (DCFH-DA) for 30 min at 37 °C. After washed 3 times with PBS, cells were added with cell lysate and the fluorescence intensity was detected at 488 nm excitation and 525 nm emission using microplate reader with fluorescence detection (ECCIPSE 50, Nicon, Japan) [11]. For NO detection, 50 μL supernatant of HMF-treated RAW264.7 cells was collected, then 50 μL Griess Reagent I and 50 μL Griess Reagent II were added and incubated for 20 min. Finally, the absorbance of the solution was measured with a microplate reader (Pennsylvania, USA) at 540 nm.

#### Detection of M1 macrophages cytokines

RAW264.7 cells were treated with LPS (1 μg/mL) or different concentrations of HMF for 24 h, the levels of TNF-*α*, IL-1*β*, IL-6 and IL-12 p70 was measured using commercial kits according to the manufacturer’s instructions. In brief, 100 μL supernatant of HMF-treated RAW264.7 cells were collected and reacted with 50 μL biotinylated antibody in the antibody-coated plates for 90 min at 37 °C. Then the microplates were washed and incubated with 100 μL streptavidin-HRP for 30 min at 37 °C. 3,3′,5,5′-Tetramethylbenzidine (TMB) was added and reacted for 20 min. The absorbance of the solution was measured with a microplate reader (Pennsylvania, USA) at 450 nm.

#### Flow cytometry assay for M1/M2 macrophage polarization in RAW264.7 cells

RAW264.7 cells were treated with LPS (1 μg/mL) or different concentrations (15.6–125 μg/mL) of HMF. After incubation for 24 h, RAW264.7 cells were collected and blocked with PBS containing 5% BSA for 15 min. Flow cytometry buffer (PBS containing 1% BSA) was then added to stop blocking. RAW264.7 cells were stained with PE-conjugated anti-CD40 antibody. Furthermore, RAW264.7 cells were stained with APC-conjugated anti-mouse CD86 antibody or APC-conjugated anti-mouse CD206 antibody to detect M1- or M2-polarized macrophages, respectively, using Aria FACS flow cytometry system (Beckman Coulter MoFlo XDP, Fullerton, CA, USA).

#### Immunofluorescence staining for M1 macrophage polarization in RAW264.7 cells

RAW264.7 cells were treated with LPS (1 μg/mL) or different concentrations (15.6–125 μg/mL) of HMF. After incubation for 24 h, RAW264.7 cells were fixed in 4% paraformaldehyde for 10 min and blocked with PBS containing 5% BSA for 15 min, then incubated with APC-conjugated anti-mouse CD86 antibody at room temperature for 20 min. The nucleus was stained by DAPI solution. After washing with PBS for twice, the specimens were imaged using a confocal laser scanning microscope (CLSM, Olympus, Tokyo, Japan).

### Statistical analysis

Statistical analysis of data was performed by unpaired two-tailed Student’s *t*-test where appropriate was used to compare two independent groups. All statistical analyses were performed using GraphPad Prism 7 (GraphPad Software, San Diego, CA). * *P* < 0.05, and ** *P* < 0.01 was considered significant.

## Results

### HPLC fingerprints of HMF for quality control

**Fig. 1:**
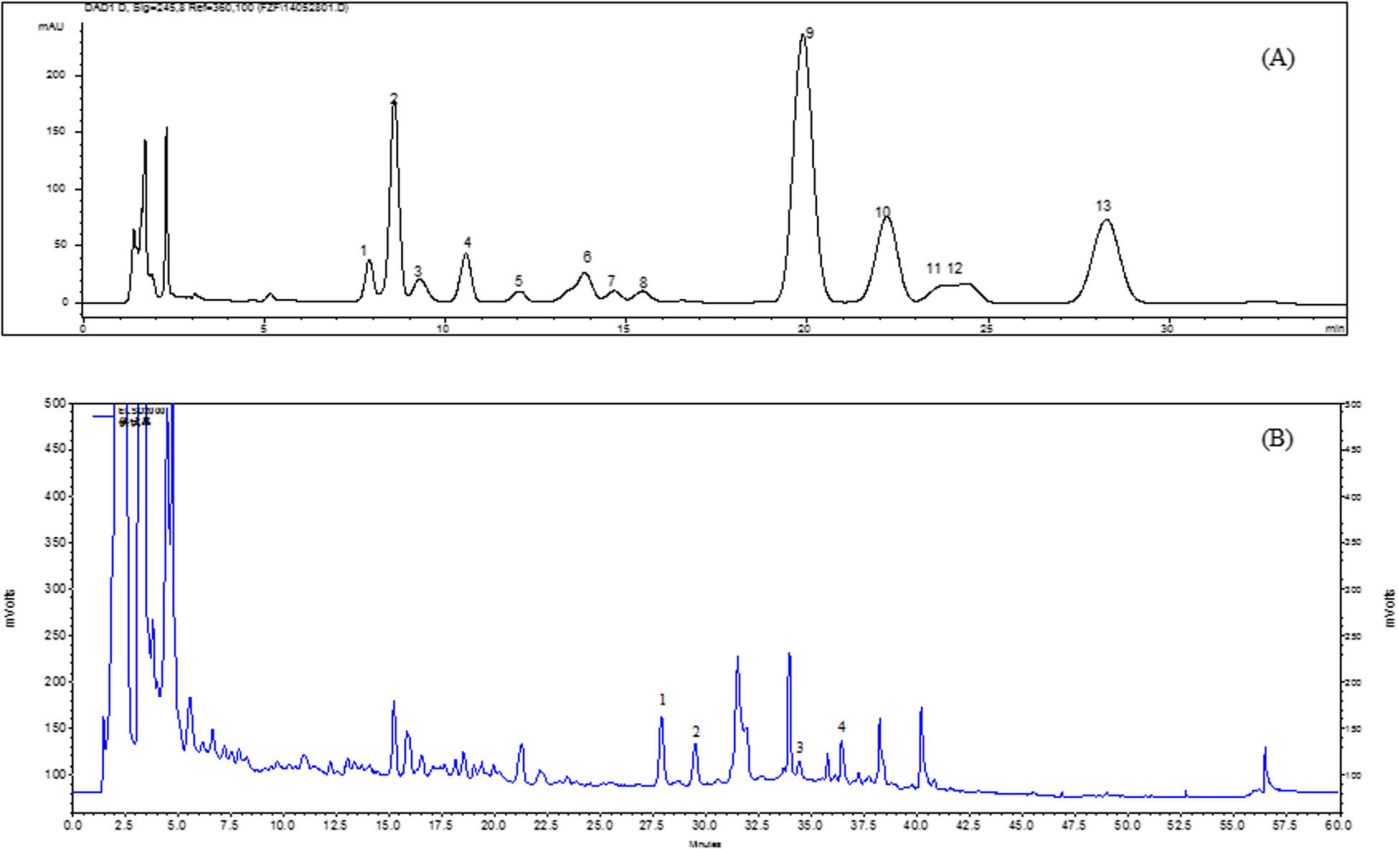
Chemical profiles of Haimufang decoction (HMF) extract. **a** PMP-HPLC for hydrophilic profile, 12 monosaccharides were: 1. GulA, 2. ManA, 3. Man, 4. GlcN, 5. Rha, 6. GlcA, 7. GalA, 9. Glc, 10. Gal, 11. Xyl, 12. Arb, 13. Fuc; and **b** HPLC-ELSD for lipophilic profile, 4 alkaloids were: 1. Solasonine, 2. Solamargine, 3. Daurisoline, 4. Dauricine

In the HMF extract, 12 monosaccharides were identified as guluronic acid (GulA), mannuronic acid (ManA), mannose (Man), glucosamine (GlcN), rhamnose (Rha), glucuronic acid (GlcA), galacturonic acid (GalA), glucose (Glc), galactose (Gal), xylose (Xyl), arabinose (Arb), and fucose (Fuc) through compared with standard using HPLC analysis (Fig. 1a). Glc, Gal, Fuc, and ManA were the most abundant monosaccharide. The content of fucoidan (derived from *Sargassum fusiforme*) in HFM was 2.64% measured with L-fucose as a standard according to our previous established PMP-HPLC method [12]. Alkaloids including dauricine and daurisoline, solasonine and solamargine in HMF were identified by comparing with standards using HPLC-ELSD method (Fig. 1b).

### Directly antiproliferative effect of HMF on lung cancer cells

#### HMF inhibits the proliferation of lung cancer cells

**Fig. 2:**
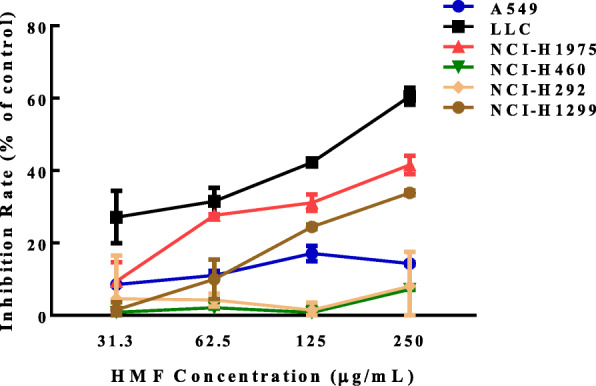
Inhibition of HMF on lung cancer cells. The inhibition rates of lung cancer cells treated with HMF (31.3–250 μg/mL) for 72 h were determined using 3-(4,5-dimethylthiazol-2-yl)-2,5-diphenyltetrazolium bromide (MTT) assay. Data are presented as mean ± SD for three independent experiments

We first investigated the cytotoxicity of HMF to various lung cancer cell lines using MTT method. As shown in Fig. 2, at the concentration ranged from 31.3 to 250 μg/mL, HMF showed cytotoxicity to various lung cancer cell lines, including LLC, NCI-H1975, and NCI-H1299, in a concentration-dependent manner, while no obvious effect against A549, NCI-H460, and NCI-H292 cell lines. As a positive control, doxorubicin (1 μM) showed more significant inhibition against LLC (95%), NCI-H1975 (90.82%), and NCI-H1299 (88.22%), A549 (92.87%), NCI-H460 (94.88%), and NCI-H292 (89.95%). And we found human lung cancer cell line NCI-H1975 was the most sensitive cells to HMF and subsequently this cell line was used to evaluate the effect of HMF on cell cycle and apoptosis.

#### HMF induces S phase arrest in NCI-H1975 cells

**Fig. 3:**
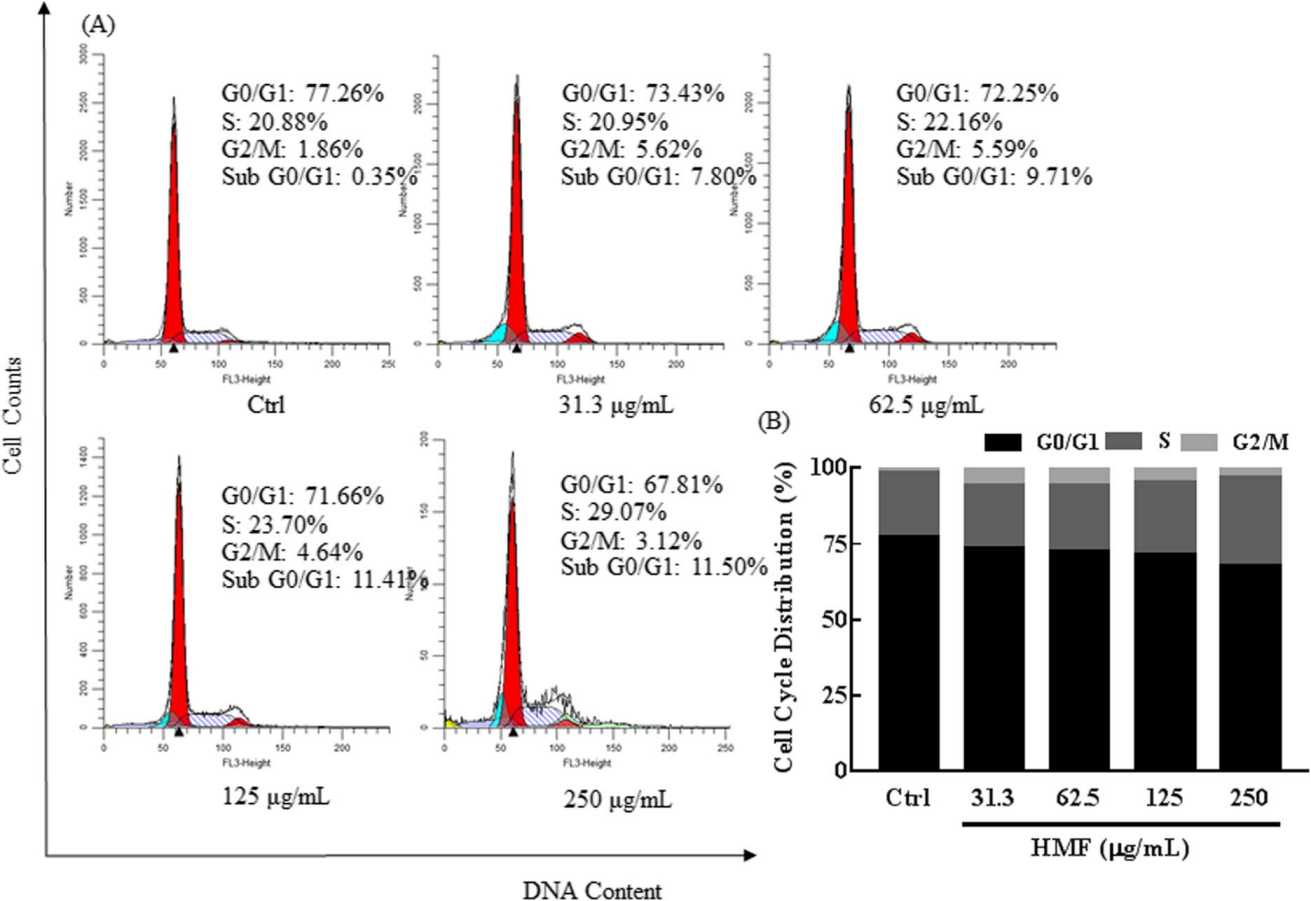
HMF increases S phase arrest in NCI-H1975 cell cycle distribution. **a** NCI-H1975 cells were exposed to different concentrations HMF (31.3–250 μg/mL) for 48 h. After treatment, the cells were collected, washed, fixed, and stained by propidium iodid (PI). Then, the cell cycle distribution was detected by flow cytometry, and the data were analyzed with ModFit LT software. Different colors were used to distinguish the peaks corresponding to cells in apoptosis, G0/G1, S, and G2/M phase. **b** The bar graph depicts the percentage of each cell cycle phase of NCI-H1975 cells in the absence or presence of HMF. All experiments were performed in three replicates

We next analyzed the effect of HMF on the cell cycle distribution of NCI-H1975 cells, and our result showed HMF induced S phase arrest in NCI-H1975 cells in a concentration-dependent manner, and also there was a decrease in the percentage of cells in G0/G1 phase (Fig. 3a). The cell percentage of S phase in the control group was 20.88%, and it increased to 22.16, 23.70, and 29.28%, when treated with 62.5, 125, and 250 μg/mL HMF (Fig. 3b), respectively, indicating HMF induced S phase arrest moderately.

When we detected the cell cycle distribution of NCI-H1975 cells, we also found that the sub-G0/G1 phase cells appeared after HMF treatment (Fig. 3a). The percentage of sub-G0/G1 cells in the control group was 0.35%, and it increased to 7.85, 9.71, 11.41, and 11.50% after treatment with HMF (31.3–250 μg/mL), indicating HMF possibly induced apoptosis in NCI-H1975 cells.

#### HMF induces apoptosis in NCI-H1975 cells

**Fig. 4:**
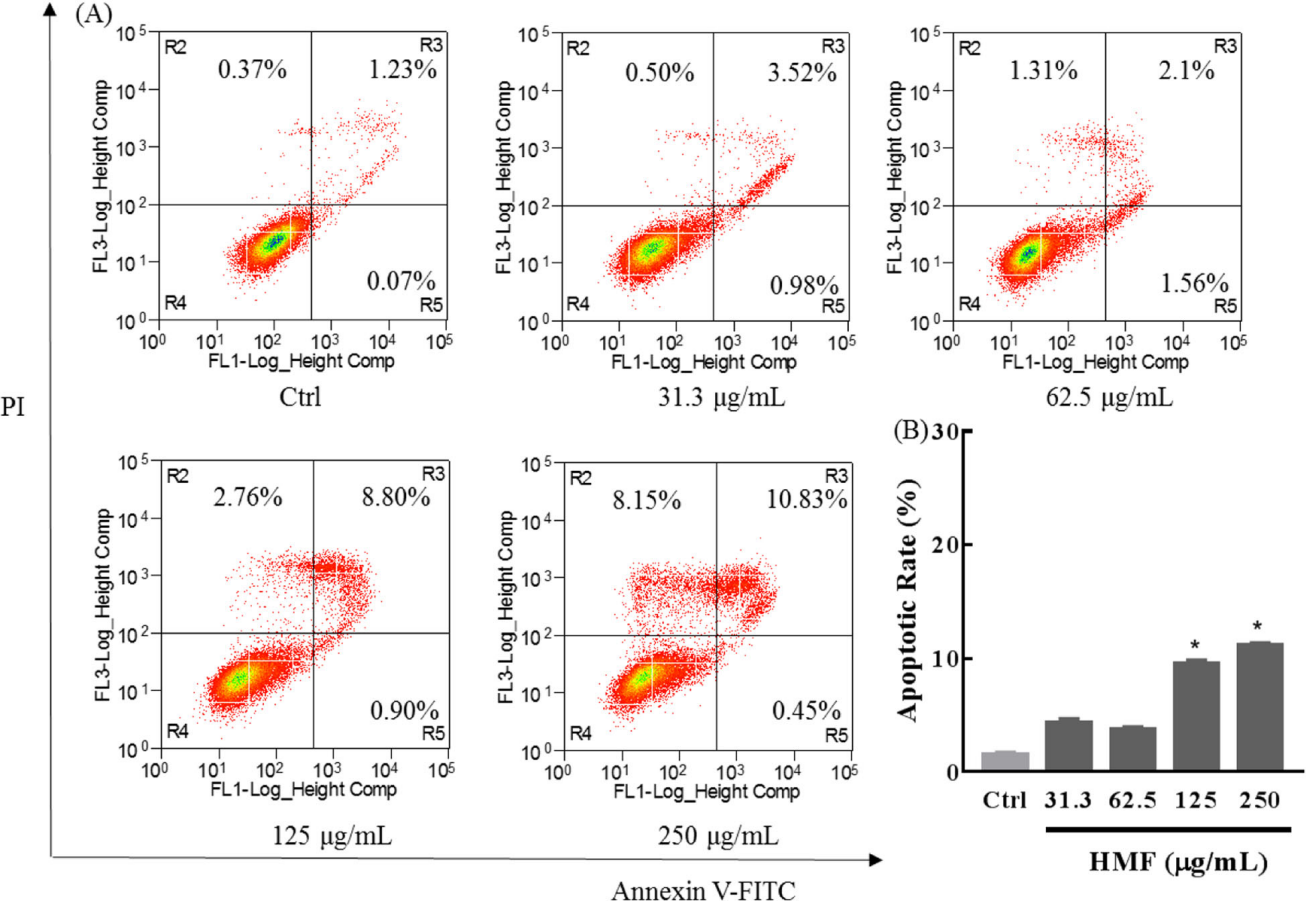
HMF triggers apoptosis in NCI-H1975 cells. **a** NCI-H1975 cells were treated with indicated concentrations of HMF (31.3–250 μg/mL) for 48 h, respectively, stained with Annexin V-fluorescein isothiocyanate (Annexin V-FITC)/PI, and determined by flow cytometry. **b** The bar graph depicts the percentage of total apoptotic cell percentage of NCI-H1975 cells in the indicated concentration of HMF. All experiments were performed in three replicates. * *P* < 0.05 was considered significant

To confirm HMF stimulated apoptosis in NCI-H1975 cells, we detected the phosphatidylserine outside of the cellular membrane by Annexin V-fluorescein isothiocyanate (Annexin V-FITC)/PI double staining assay (Fig. 4a). The total apoptotic cell percentage (the sum of R3 and R5 regions) of the control group was about 1.30%, and it increased to 4.50, 3.66, 9.70, and 11.28% after treated with HMF (31.3–250 μg/mL) for 48 h (Fig. 4b), respectively. Notably, relatively higher concentration of HMF (250 μg/mL) could also moderately increase the percentage of necrotic cells (Fig. 4a).

#### HMF regulates the expression of apoptosis-associated proteins in NCI-H1975 cells

**Fig. 5:**
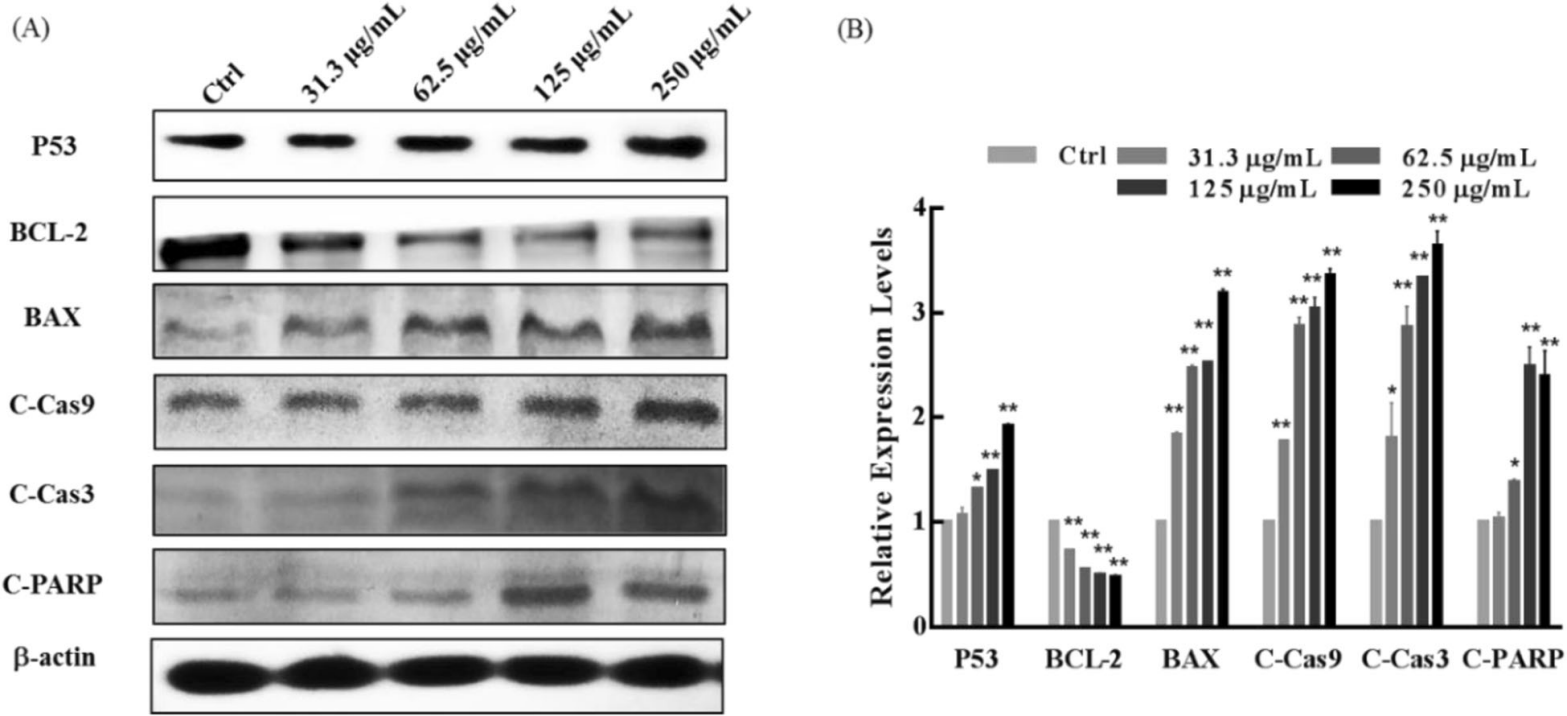
Effect of HMF on the expression of apoptosis-associated protein in NCI-H1975 cells. **a** The expression levels of apoptosis-associated proteins in NCI-H1975 cells treated with HMF (0–250 μg/mL) for 48 h were analyzed by western blotting using anti-P53, anti-BCL-2 associated X protein (BAX), anti-B-cell lymphoma-2 (BCL-2), anti-cleaved-cysteinyl aspartate specific proteinase-3 (C-Cas3), anti-cleaved-cysteinyl aspartate specific proteinase (C-Cas9), anti-cleaved-poly-adenosine diphosphate-ribose polymerase (C-PARP). **b** The results of the western blot analysis were quantified; *n* = 3. Data are expressed as the mean ± SD. * *P* < 0.05, ** *P* < 0.01 vs. control

The tumor suppressor P53 plays a crucial role in the regulation of cell cycle arrest and apoptosis [13]. In the present study, the expression of P53 significantly increased in NCI-H1975 cells when treated with HMF, in a concentration-dependent manner (Fig. 5a and b), which was consistent with the S phase cell cycle arrest and apoptosis stimulation induced by HMF. Abnormal ratio of pro-apoptotic gene BAX/anti-apoptotic gene BCL may cause apoptosis [14]. We then detected the expression level of BAX and BCL-2, and the results showed the expression of BAX was noticeably elevated, while, BCL-2 significantly decreased in NCI-H1975 cells treated with HMF (Fig. 5a and b). Furthermore, cleaving of the caspase-9/caspase-3 is a hallmark of the apoptosis, and cleaved PARP (C-PARP) is an activated substrate of cleaved caspase 3 (C-Cas3) and is a marker of apoptosis [15]. After HMF treatment, we found that C-Cas3, cleaved caspase-9 (C-Cas9), and C-PARP significantly increased (Fig. 5a and b), confirming apoptosis occurred after HMF treatment in NCI-H1975 cells.

### Immunomodulatory effect of HMF on RAW264.7 cells

#### HMF has no proliferation inhibition on RAW264.7 cells

**Fig. 6:**
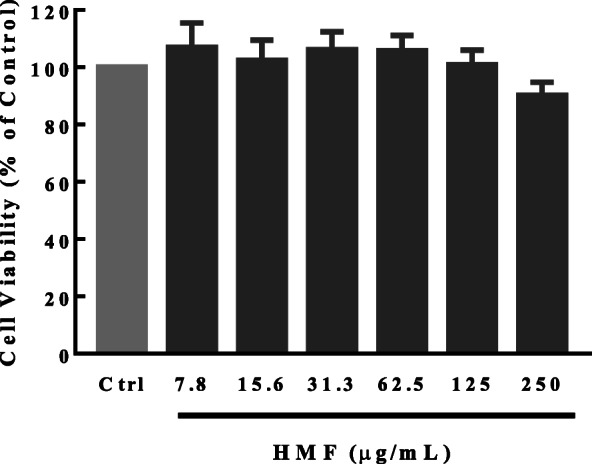
Effect of HMF on RAW264.7 cells proliferation. RAW264.7 cells were treated with HMF (7.8–250 μg/mL) for 24 h. The proliferation of RAW264.7 cells were detected by MTT assay. Data are presented as mean ± SD for three independent experiments

To evaluate whether HMF also had immunomodulatory effect, we first detected its effect on the proliferation of RAW264.7 cells, and we found HMF (7.8–250 μg/mL) showed no effect on the viability of RAW264.7 cells, while slight inhibition under the concentration of 250 μg/mL for 24 h (Fig. 6), suggesting HMF did not affect the proliferation of macrophage.

#### HMF enhances the anticancer and the phagocytosis activity of RAW264.7 cells

**Fig. 7:**
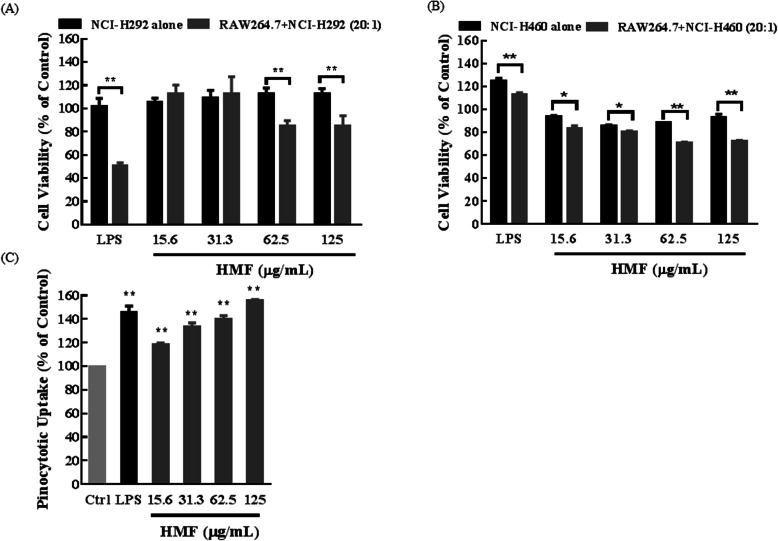
Cytotoxic and phagocytosis activities of HMF-treated RAW264.7 cells. RAW264.7 cells were first treated with HMF (15.6–125 μg/mL) for 24 h, then lung cancer NCI-H460 (**a**) and NCI-H292 (**b**) cells were added. Then, MTT assay was performed, * *P* < 0.05, ** *P* < 0.01 versus the lung cancer cells alone group. **c** HMF promotes the phagocytosis of RAW264.7 cells. After treated with HMF (15.6–125 μg/mL) for 24 h, the medium was replaced with neutral red physiological saline. The plates were washed and pyrolysis liquid was added. The absorbance at 570 nm was measured. * *P* < 0.05, ** *P* < 0.01 versus the control group

When activated, the macrophages show increased antiproliferation against targeted cells [16]. To investigate the activation of HMF on RAW264.7 cells, the antiproliferative activities of HMF-treated RAW264.7 cells were determined. Positive control LPS (macrophages activator) significantly enhanced the inhibition activity of RAW264.7 cells on co-cultured lung cancer cells. Similarly, HMF (15.6–125 μg/mL) also remarkably enhanced the antiproliferative activity of RAW264.7 cells on co-cultured NCI-H460 cells (Fig. 7a) and NCI-H292 cells (Fig. 7b) in a concentration-dependent manner, indicating HMF activated RAW264.7 cells.

Next, we detected the effect of HMF on RAW264.7 cells phagocytosis using neutral red assay. As shown in Fig. 7c, as the positive control LPS did, HMF (15.6–125 μg/mL) significantly enhanced the phagocytosis activity of RAW264.7 cells in a concentration-dependent manner (*P* < 0.01), suggesting HMF enhancing the phagocytosis ability of RAW264.7 cells to inhibit tumor cell proliferation.

#### HMF activates RAW264.7 cells and enhances the secretion of macrophage factors

**Fig. 8:**
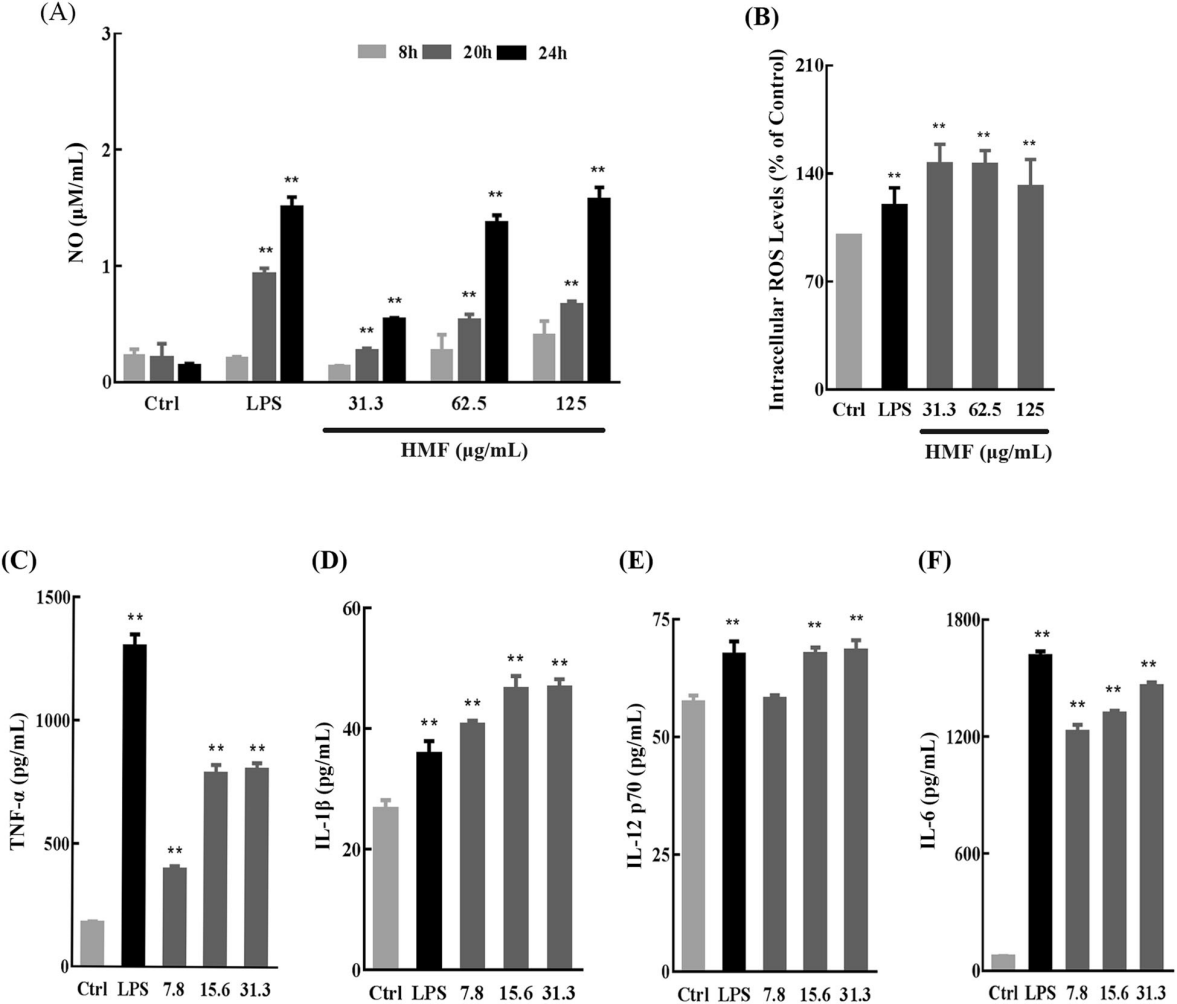
HMF promotes total nitric oxide (NO), intracellular reactive oxygen species (ROS), and macrophage factors production of RAW264.7 cells. **a** RAW264.7 cells were treated with LPS (1 μg/mL) or HMF (31.3, 62.5, 125 μg/mL) for 8, 20, and 24 h. The releases of NO were determined by using NO assay kit. **b** RAW264.7 cells were treated with LPS (1 μg/mL) or HMF (31.3, 62.5, 125 μg/mL) for 24 h. The intracellular ROS was detected by ROS assay kit. For the cytokines assay, RAW264.7 cells were treated with LPS (1 μg/mL) or HMF (7.8, 15.6, 31.3 μg/mL) for 24 h. Tumor necrosis factor-*α* (TNF-*α*) (**c**), interleukin-1*β* (IL-1*β*) (**d**), interleukin 12 p70 (IL-12 p70) (**e**), interleukin 6 (IL-6) (**f**) were all measured with enzyme linked immunosorbent assay kit. Each experiment was performed in triplicate, and values were represented as means ± SD. * *P* < 0.05, and ** *P* < 0.01 versus the control

We then detected the activation related molecules in RAW264.7 cells after HMF treatment. As shown in Fig. 8a, like the positive control LPS, HMF promoted NO production in a concentration- and time-dependent manner in RAW264.7 cells. As shown in Fig. 8b, compared with the control group, HMF promoted ROS production in a concentration-dependent manner in RAW264.7 cells. Like LPS stimulation in RAW264.7 cells, HMF also resulted in significant increase in the secretion level of macrophage factors, including TNF-*α* (Fig. 8c), IL-1*β* (Fig. 8d), IL-12 p70 (Fig. 8e), and IL-6 (Fig. 8f) in a concentration-dependent manner, indicating HMF activated RAW 264.7 cells.

#### HMF induces M1 macrophage polarization in RAW264.7 cells

**Fig. 9:**
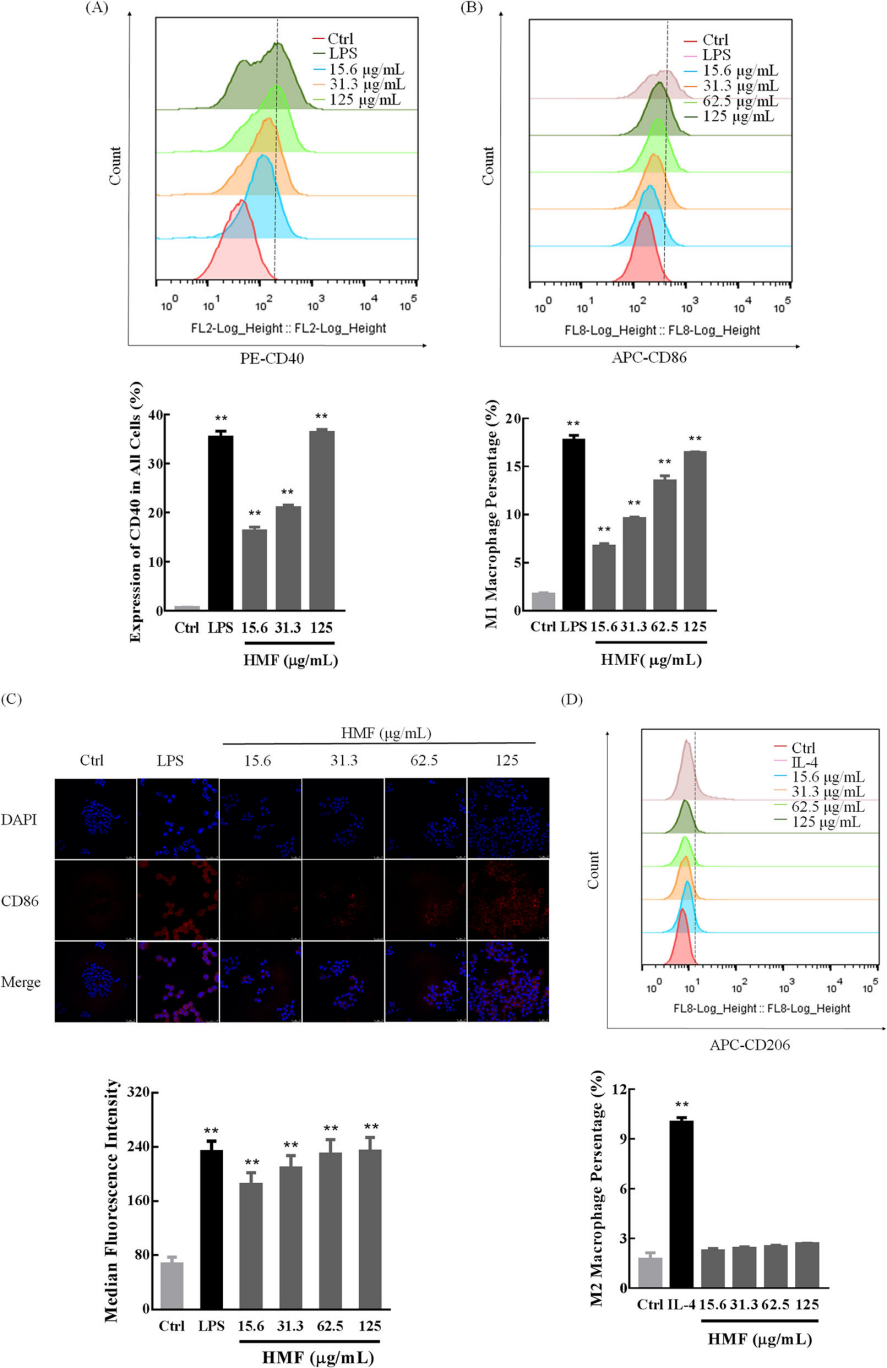
The activation and polarization of RAW264.7 cells in the presence of and absence of HMF. RAW264.7 cells were treated with LPS (1 μg/mL) or IL-4 (10 ng/mL) or different concentrations (15.6–125 μg/mL) of HMF for 24 h, and then incubated with the indicated antibodies. The expression of macrophage activation marker-cluster of differentiation (CD) 40 (**a**), M1 phenotype signature marker CD 86 (**b**), and M2 phenotype signature marker CD 206 (**d**) were detected by Aria FACS flow cytometry system (Beckman Coulter MoFlo XDP, Fullerton, CA, USA). Immunofluorescence staining of CD86 (red) (**c**) on macrophages and the nuclei stained with DAPI (blue) were imaged using a confocal laser scanning microscope (CLSM, Olympus, Tokyo, Japan) (magnification × 200). Quantitative analysis of the relative median fluorescence intensity of CD86 by Image-Pro Plus software. Different colors were used to distinguish the peaks corresponding to different groups. Each experiment was performed in triplicate, and values were represented as means ± SD. * *P* < 0.05, and ** *P* < 0.01 versus the control

To further determine the activation of RAW 264.7 cells, macrophage activation marker CD40 was detected. As shown in Fig. 9a, like the positive control LPS, CD40 expression increased remarkably in a concentration-dependent manner in RAW 264.7 cells when treated with HMF for 24 h (*P* < 0.01), which confirmed that HMF could activate RAW264.7 cells.

Subsequently, we detected the macrophages differentiation by flow cytometry and immunofluorescence assay. Compared with the control group cells, M1 phenotype maker CD86 expression increased significantly in RAW264.7 cells when treated with different concentration of HMF (Fig. 9b, *P* < 0.01). The immunofluorescence staining also confirmed that the expression of CD86 in HMF-incubated RAW264.7 cells elevated in a concentration-dependent manner (Fig. 9c, *P* < 0.01). However, no significant difference of the expression of M2 phenotype CD206 was found in different concentration HMF group (Fig. 9d, *P* > 0.05).

In summary, our results indicated that HMF inhibited the proliferation of lung cancer cells by arresting cell cycle and inducing apoptosis. Moreover, we investigated the activation activity of HMF on RAW264.7 macrophage cells and found HMF activation and improved phagocytosis of RAW264.7 cells by polarizing RAW264.7 cells to M1 phenotype.

## Discussion

This study aims to elucidate the possible mechanisms of HMF for the treatment of lung cancer. Based on the characteristics of the multiple action mechanisms of TCM, we first detected the cytotoxic effect of HMF on lung cancer cells, and the results indicated HMF showed cytotoxicity to various lung cancer cell lines, including LLC, NCI-H1975, and NCI-H1299, suggesting HMF showed certain selectivity for different lung cancer cell lines. As LLC cells are mouse obtained cancer cells, we chose the most sensitive human lung cancer cell line NCI-H1975 in the further studies evaluating the cell cycle arrest, and apoptosis after HMF treatment. NCI-H1975 cell line is an epidermal growth factor receptor (EGFR) mutated cell line [17], while NCI-H1299, A549, and NCI-H460 are RAS mutant type [18]. Whether the selectivity of HMF to NCI-H1975 cell line has a relationship with the mutant type remains further investigations.

Cell cycle arrest usually induces cell proliferation inhibition [19]. To explore the mechanisms underlying the antiproliferative activity of HMF in NCI-H1975 cells, we then detected the cell cycle distribution. Like the cell cycle arrest effect of some TCM [20–22], HMF could induce S phase cell cycle arrest. Sub-G0/G1 cells are the hallmark of apoptosis, and the flow cytometry results also confirmed the apoptosis inducing effect of HMF. Interestingly, HMF induced necrosis at relative higher concentration, which was consistent with the chemotherapeutic agent cisplatin that also stimulates necrosis in cancer cells [23]. It has been reported that inducing necrosis of tumor tissues could suppress tumor growth in mouse xenograft bladder cancer models [24], so we speculated necrosis induction of HMF might also be responsible for antiproliferative activity of HMF. Apoptosis is a death process involving a series of apoptosis-related proteins. To further confirm HMF induced apoptosis in NCI-H1975 cells, we detected the apoptosis-related proteins. The tumor suppressor P53 plays a crucial role in the regulation of cell cycle arrest and apoptosis [13]. Considering P53 can induce BAX transcription [25], and abnormal BAX/ BCL ratio, may cause apoptosis [14]. Caspase-9 and caspase-3, the important effector caspases of the apoptosis pathways, are downstream proteins of the BCL family. Cleaving of the caspase-9/caspase-3 is a hallmark of the apoptosis. C-PARP is an activated substrate of C-Cas3 and an early marker of apoptosis [15]. Western blot results indicated that HMF could promote NCI-H1975 cells apoptosis by up-regulating pro-apoptotic gene and down-regulating anti-apoptotic gene expression. Thus, both cell cycle arrest and apoptosis induction contributed to the proliferation inhibition of HMF against NCI-H1975 cells.

However, it should be noted that both the cell cycle arrest, apoptosis induction, as well as the necrosis stimulation induced by HMF were moderate, which was not consistent with its strong clinical effect, suggesting the direct inhibitory effect against the cancer cells was only a part of its multiple anticancer mechanisms and other mechanisms also existed. Water-soluble components of HMF contained fucoidan (derived from *Sargassum fusiforme*), dauricine and daurisoline (derived from *Mertispermum dauricum*), solasonine and solamargine (derived from *Solanum nigrum*). Dauricine [26], daurisoline [27], solasonine [28] and solamargine [29] could inhibit proliferation of cancer cells by inducing cell cycle arrest and apoptosis. Interestingly, a moderate degree of cytotoxicity of HMF on lung cancer cells might be a result of low concentration of dauricine and daurisoline, solasonine and solamargine tested by HPLC-ELSD method. Fucoidan is a unique sulfate-containing polysaccharide in sargassum. The structure of fucoidan is closely related to the species, which causes different activities. Fucoidan not only shows weak cytotoxic activity in vitro [30], but also prolongs the survival rate of experimental animals and exhibits good anticancer activities in vivo by inducing apoptosis, enhancing immune function, and inhibiting angiogenesis [31]. Moreover, it has been reported that *Solanum nigrum* [32] and *Mertispermum dauricum* alkaloids [27] can significantly inhibit the proliferation of tumor cells in vitro, and oyster polysaccharide [33] can enhance the secretion of immune factors and increase the thymus index and spleen index of tumor-bearing mice.

It has been reported that immune system regulation is one of the mechanisms for the anticancer activity of some Chinese medicine formulas [34]. Therefore, we next tried to explore the immunomodulatory effect on RAW264.7 cells of HMF. Macrophages co-culture system is usually used to detect the effect of effector cells on target cells [35]. Up to 125 μg/mL, HMF had negligible effect on proliferation of RAW264.7 cells, NCI-H460 cells, and NCI-H292 cells, so we chose this concentration of HMF, NCI-H460 cells and NCI-H292 cells to avoid the direct cytotoxic effect of HMF on target cells or effector cells. To investigate whether HMF could activate RAW264.7 cells and attenuate tumor cell growth, RAW264.7 cells were treated with HMF and then the HMF-treated RAW264.7 cells were co-cultured with NCI-H460 cells and NCI-H292 cells. The results showed HMF-treated RAW 264.7 cells could inhibit proliferation of NCI-H460 cells and NCI-H292 cells more efficiently than the non-treated RAW 264.7 cells, which indicated HMF might activate macrophages. Macrophage phagocytosis is considered as a part of activated immune responses to cancer cells [36]. To explore the mechanisms underlying the enhanced antiproliferative activity of RAW264.7 cells against lung cancer cells, we detected the effects of HMF on phagocytosis by neutral red, the results confirmed that HMF enhanced the phagocytosis of macrophages.

It has been reported that macrophages exerted anticancer effects by secreting NO, intracellular ROS, and macrophage factors [15]. In addition, ROS serves as secondary messengers in signaling transduction during macrophages phagocytosis [37]. In our present study, HMF promoted NO production and ROS production in RAW264.7 cells, also suggesting RAW264.7 cells were activated. TNF-*α*, IL-1*β*, IL-6 and IL-12 p70 are key cytokines in immunity, and are indispensable to the macrophages functions [17]. HMF also resulted in significant increase in the secretion level of macrophage factors. These results further confirmed that HMF promoted the activation of RAW264.7 cells.

Activated macrophages can differentiate into M1 phenotype possessing tumor inhibition activity, or M2 phenotype possessing tumor promotion activity [38]. Flow cytometry and immunofluorescence results of CD40, CD86, and CD206 expression further showed HMF could activate macrophage, and cause M1 phenotype polarization without affecting the M2 phenotype. It has been reported that oyster polysaccharide could enhance the secretion of immune factors and increase the immunity of tumor-bearing mice [33], and Chen et al. also reported that polysaccharides of *Flammulina Velutipes* stipe activated macrophage and showed obvious anticancer activities [39]. We deduced that the polysaccharide fraction in HMF might contribute to immunomodulatory effect on RAW 264.7 macrophage cells. However, further experiments are needed to confirm this hypothesis and identify the polysaccharide fraction in HMF responsible for the immunomodulatory effect. It should be noted that we used RAW264.7 cells, which are mouse macrophages, to evaluate the immunomodulatory effect of HMF. Previous studies have shown that these cells are not reliable as indicators of human immune response [40], only be used as a preliminary investigation of the immunomodulatory effects. Therefore, human M1 and M2 macrophages will be used in our further works related to the immunomodulatory effects of HMF to solid our present findings.

It seems that the apoptosis induction on cancer cells and the immunomodulatory effect on macrophage cells of HMF is parallel, and both contributes to the anticancer activity of HMF. Similarly, some reports also showed that some Chinese medicine formulas also revealed both apoptosis induction activity and immunomodulatory effect, however, up to now, it seems that there is no direct relationships between the apoptosis induction and immunomodulatory effect [41–43]. In the case of HMF, whether there is a direct connection between the apoptotic mechanisms of HMF on lung cancer and immunomodulatory effect on RAW264.7 cells needs further more evidence. Moreover, pharmacokinetics investigation are needed in the future to reveal the effective concentration in the blood plasma to guide clinical HMF administration.

## Conclusions

In summary, HMF is a patented clinical prescription of TCM for lung cancer, and the mechanisms underlying the anticancer activity is mainly by inhibiting proliferation, arresting cell cycle, inducing apoptosis in NCI-H1975 lung cancer cells, and also by activating and stimulating M1 phenotype polarization in macrophages, which provides more scientific understanding of its anticancer activity and mechanisms.

## Supplementary information

**Additional file 1.** (PPTX 256 kb)

## Data Availability

Data are all contained within the manuscript.

## Abbreviations

GulA: Guluronic acid
ManA: Mannuronic acid
Man: mannose
GlcN: Glucosamine
Rha: Rhamnose
GlcA: Glucuronic acid
GalA: Galacturonic acid
Glc: Glucose
Gal: Galactose
Xyl: Xylose
Arb: Arabinose
Fuc: Fucose
TCM: Traditional Chinese medicine
HMF: Haimufang decoction
BCL-2: B cell lymphoma-2
BAX: B cell lymphoma-2 (BCL-2) associated X protein
C-caspase-3: cleaved-cysteinyl aspartate specific proteinase-3
C-caspase-9: cleaved-cysteinyl aspartate specific proteinase-9
C-PARP: cleaved-poly-adenosine diphosphate-ribose polymerase
C-Cas3: C-caspase-3
C-Cas9: C-caspase-9
FBS: Fetal bovine serum
NO: Nitric oxide
ROS: Reactive oxygen species
MTT: 3-(4,5-dimethylthiazol-2-yl)-2,5-diphenyltetrazolium bromide
LPS: lipopolysaccharides
DOX: Doxorubicin
BSA: Bovine serum albumin
DAPI: 4′,6-diamidino-2-phenylindole
PBS: Phosphate buffer saline
PE: Phycoerythrin
APC: Allophycocyanin
CD: Cluster of differentiation
HRP: Horseradish peroxidase
TNF-*α*: tumor necrosis factor-*α*
IL-1*β*: interleukin-1*β*
IL-12 p70: Interleukin 12 p70
IL-6: interleukin 6
LLC: mouse lung cancer cell line Lewis
NCI-H: Non-small cell lung cancer-homo sapiens
HPLC: High performance liquid chromatography
HPLC-ELSD: HPLC method with evaporative light scattering detector (ELSD)
