# Supplementary material for: Haimufang decoction, a Chinese medicine formula for lung cancer, arrests cell cycle, stimulates apoptosis in NCI-H1975 cells, and induces M1 polarization in RAW 264.7 macrophage cells
Source: BMC Complement Med Ther. 2020 Aug 5;20:243. doi: 10.1186/s12906-020-03031-1 (PMC7404932; doi:10.1186/s12906-020-03031-1)
Supplement: Supplementary file 1 — Additional file 1. (PPTX 256 kb) [file 12906_2020_3031_MOESM1_ESM.pptx]

## Slide 1
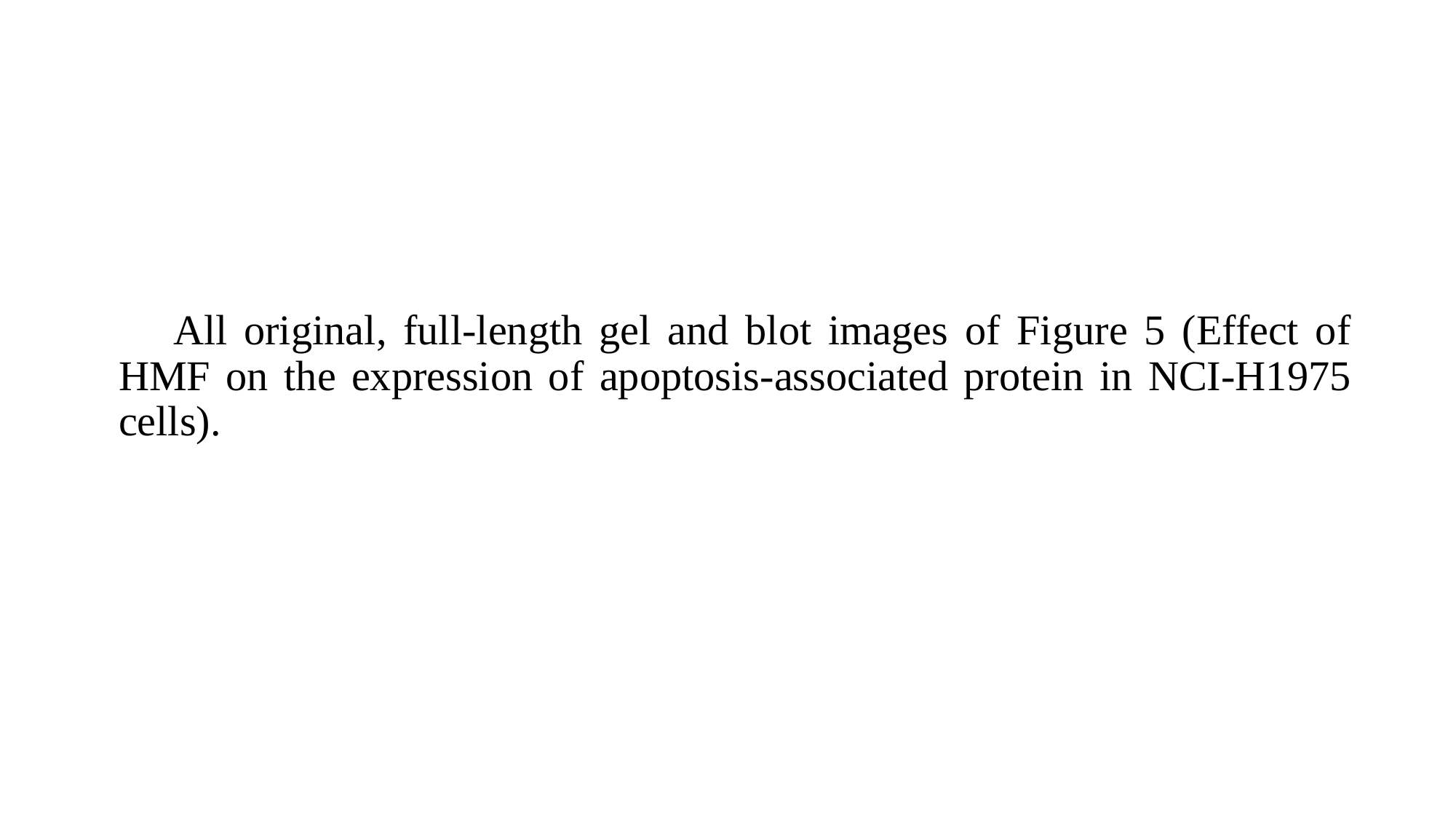

# All original, full-length gel and blot images of Figure 5 (Effect of HMF on the expression of apoptosis-associated protein in NCI-H1975 cells).

## Slide 2
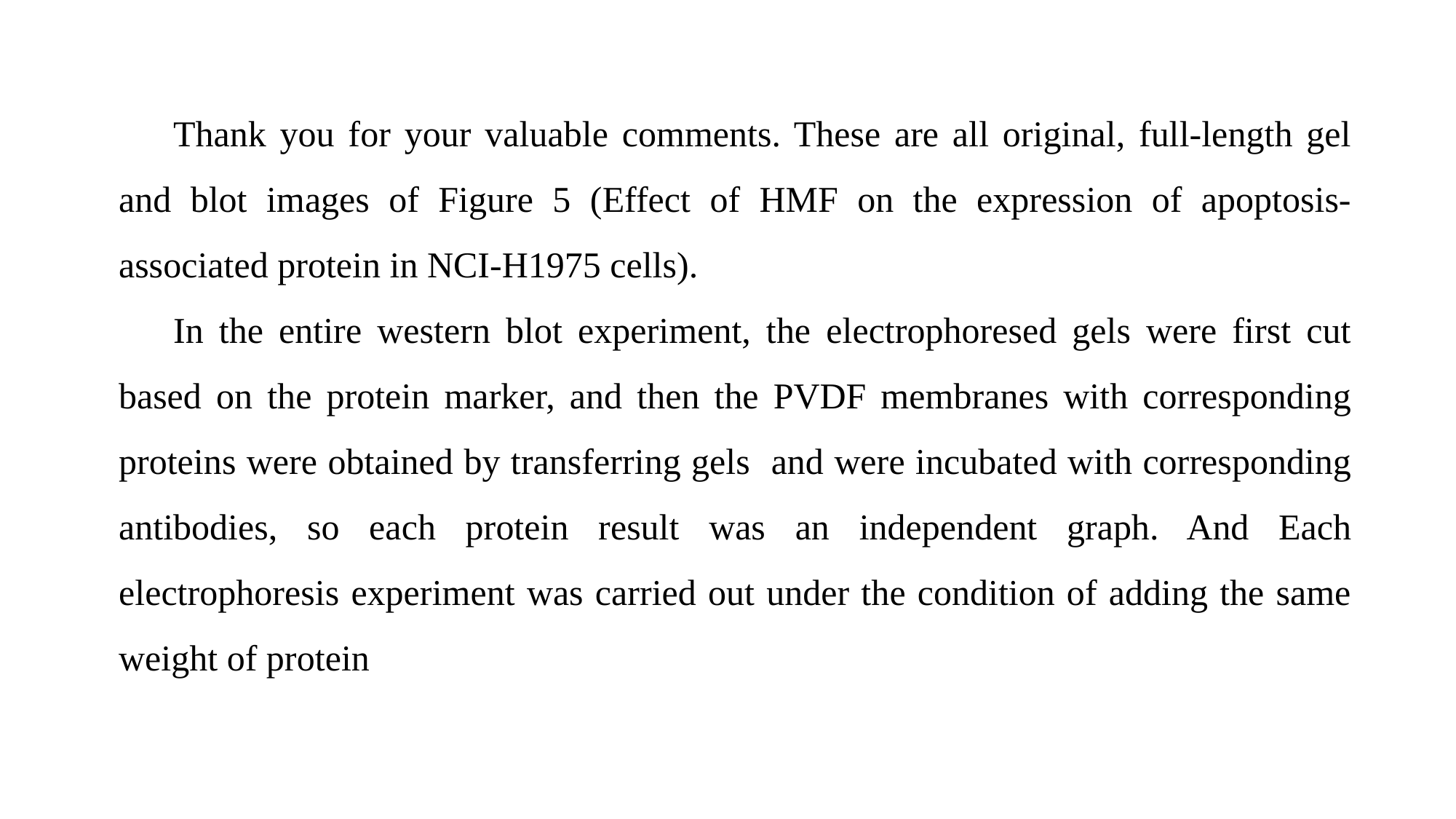

Thank you for your valuable comments. These are all original, full-length gel and blot images of Figure 5 (Effect of HMF on the expression of apoptosis-associated protein in NCI-H1975 cells).
In the entire western blot experiment, the electrophoresed gels were first cut based on the protein marker, and then the PVDF membranes with corresponding proteins were obtained by transferring gels and were incubated with corresponding antibodies, so each protein result was an independent graph. And Each electrophoresis experiment was carried out under the condition of adding the same weight of protein

## Slide 3
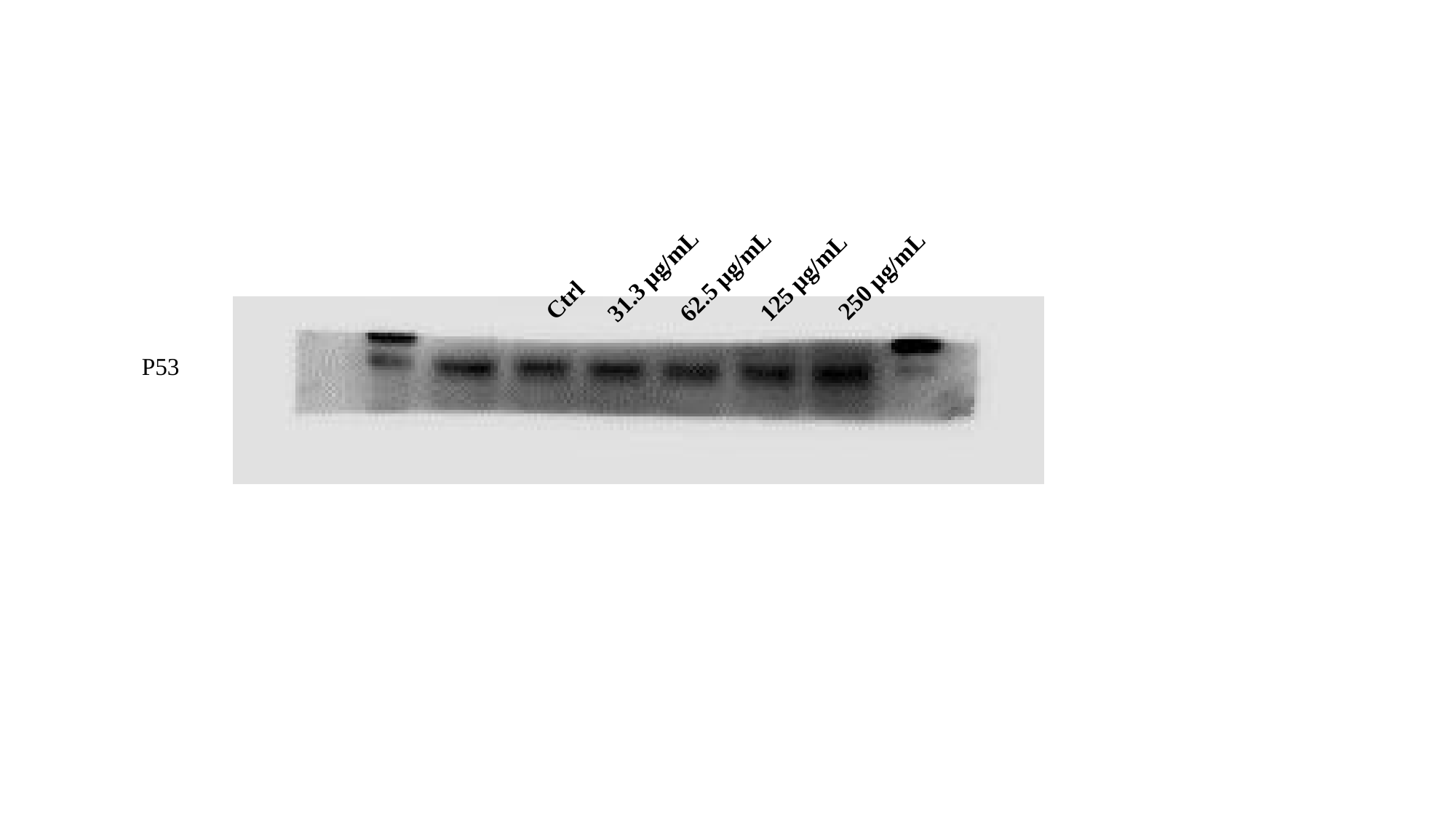

31.3 μg/mL
62.5 μg/mL
250 μg/mL
125 μg/mL
Ctrl
P53

## Slide 4
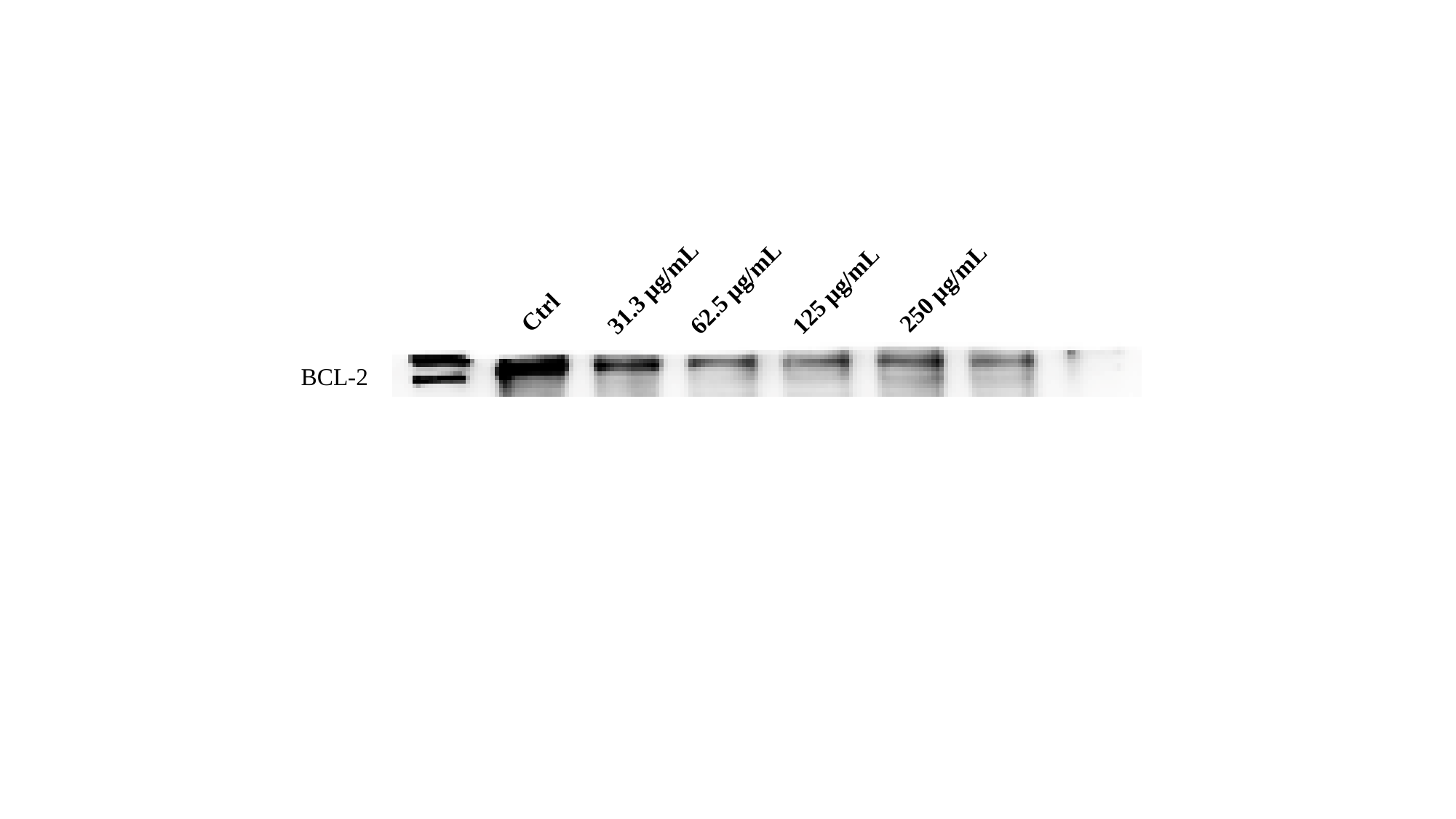

31.3 μg/mL
62.5 μg/mL
250 μg/mL
125 μg/mL
Ctrl
BCL-2

## Slide 5
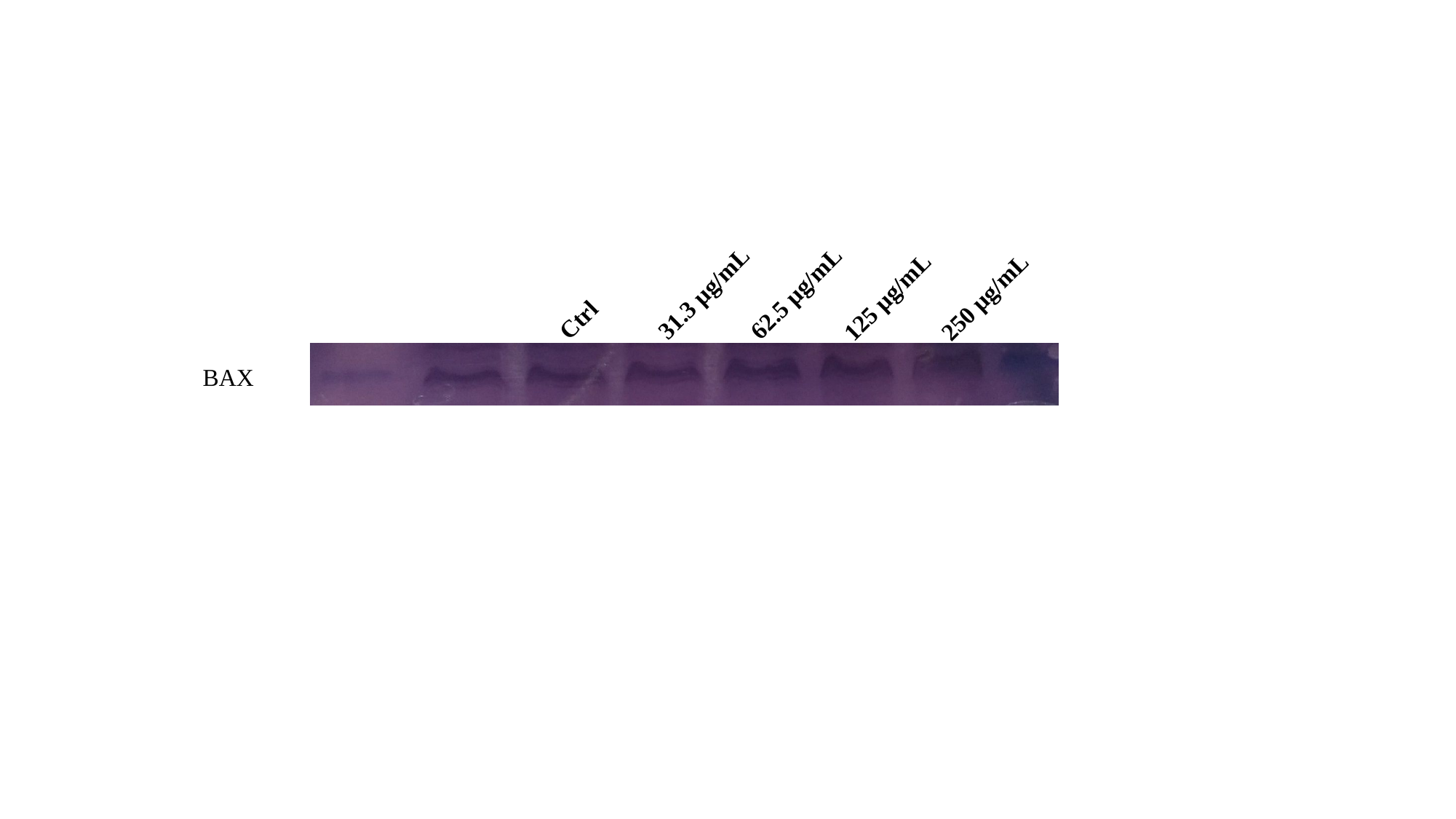

31.3 μg/mL
62.5 μg/mL
125 μg/mL
250 μg/mL
Ctrl
BAX

## Slide 6
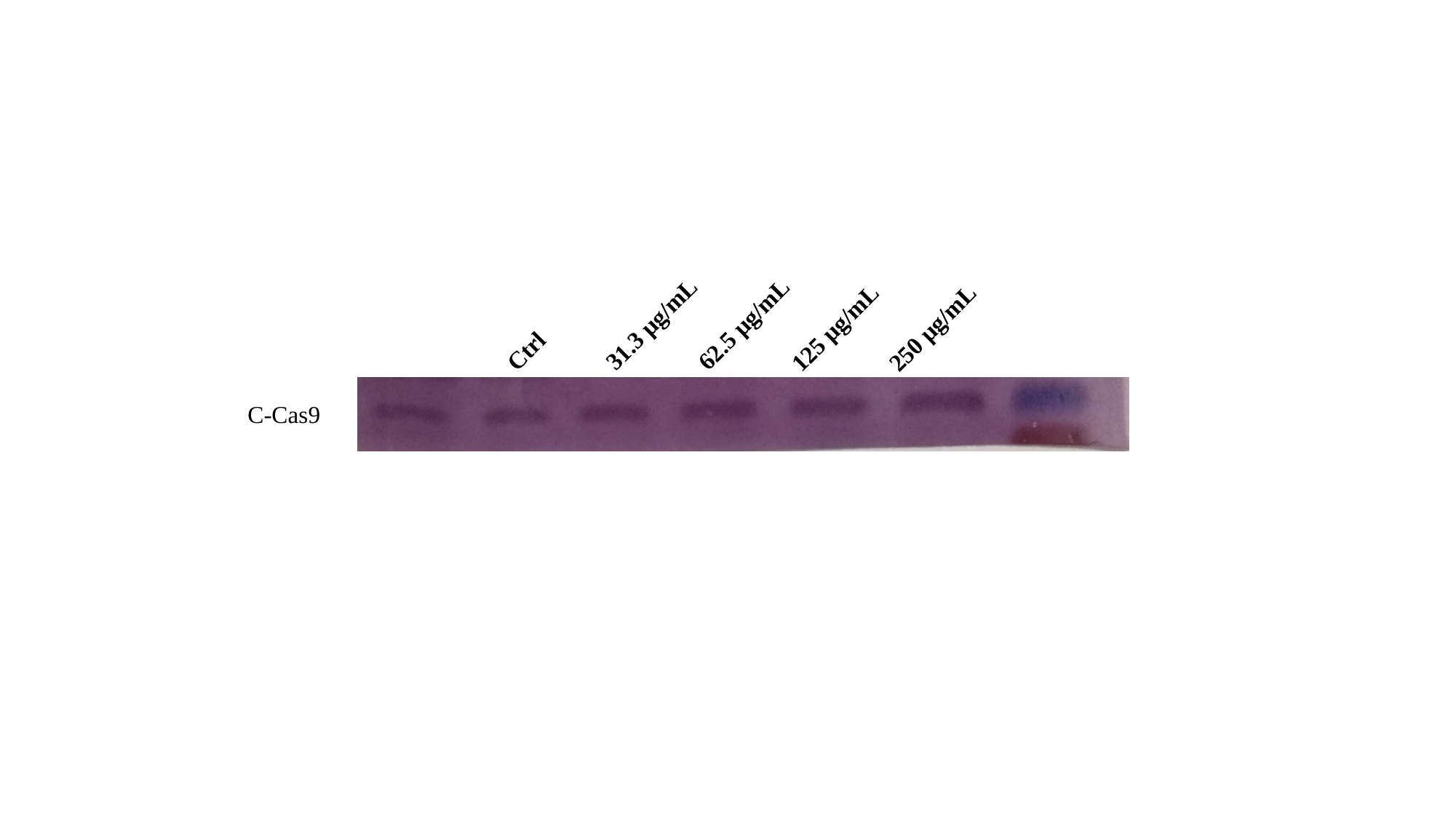

31.3 μg/mL
62.5 μg/mL
125 μg/mL
250 μg/mL
Ctrl
C-Cas9

## Slide 7
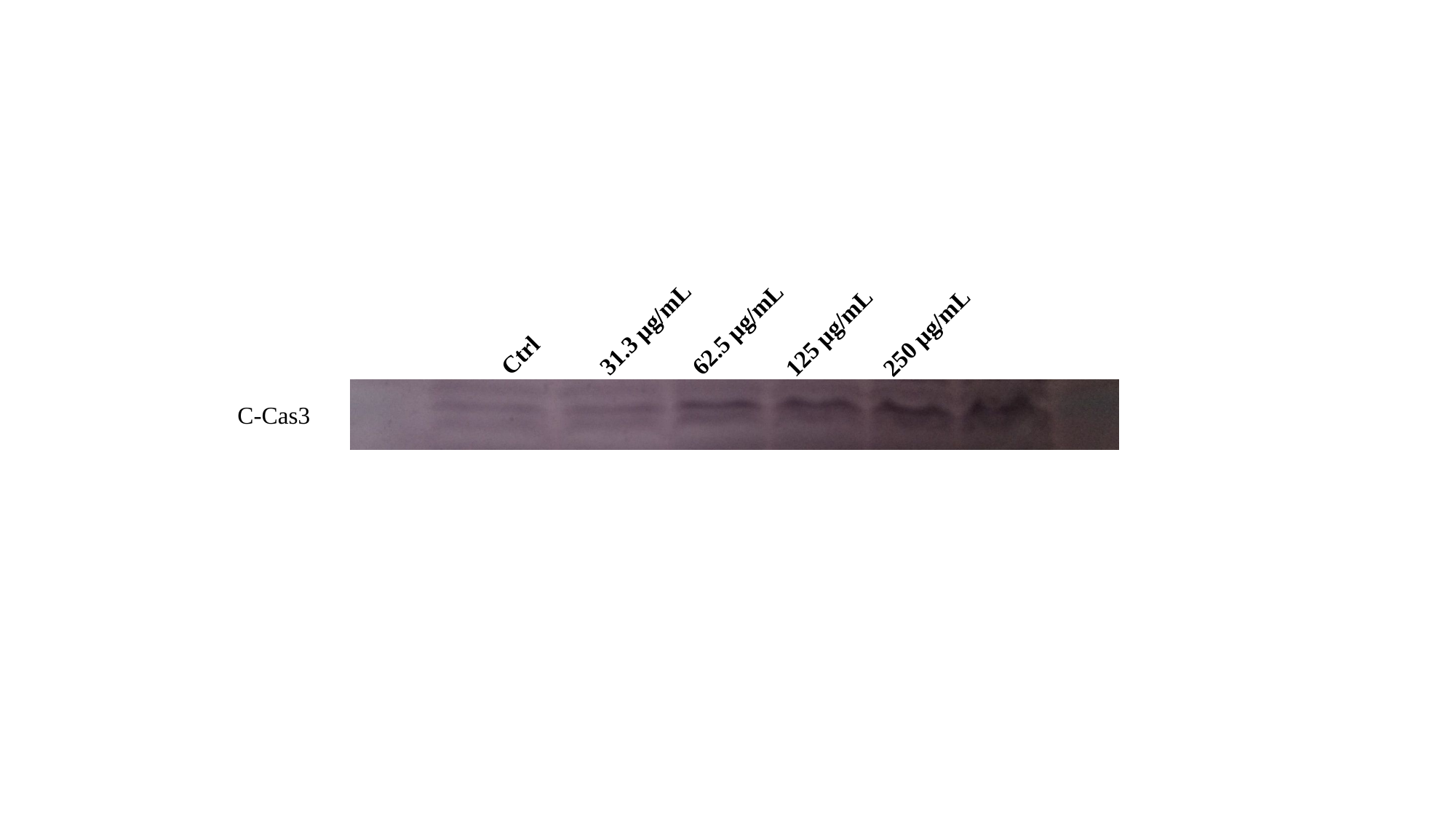

31.3 μg/mL
62.5 μg/mL
125 μg/mL
250 μg/mL
Ctrl
C-Cas3

## Slide 8
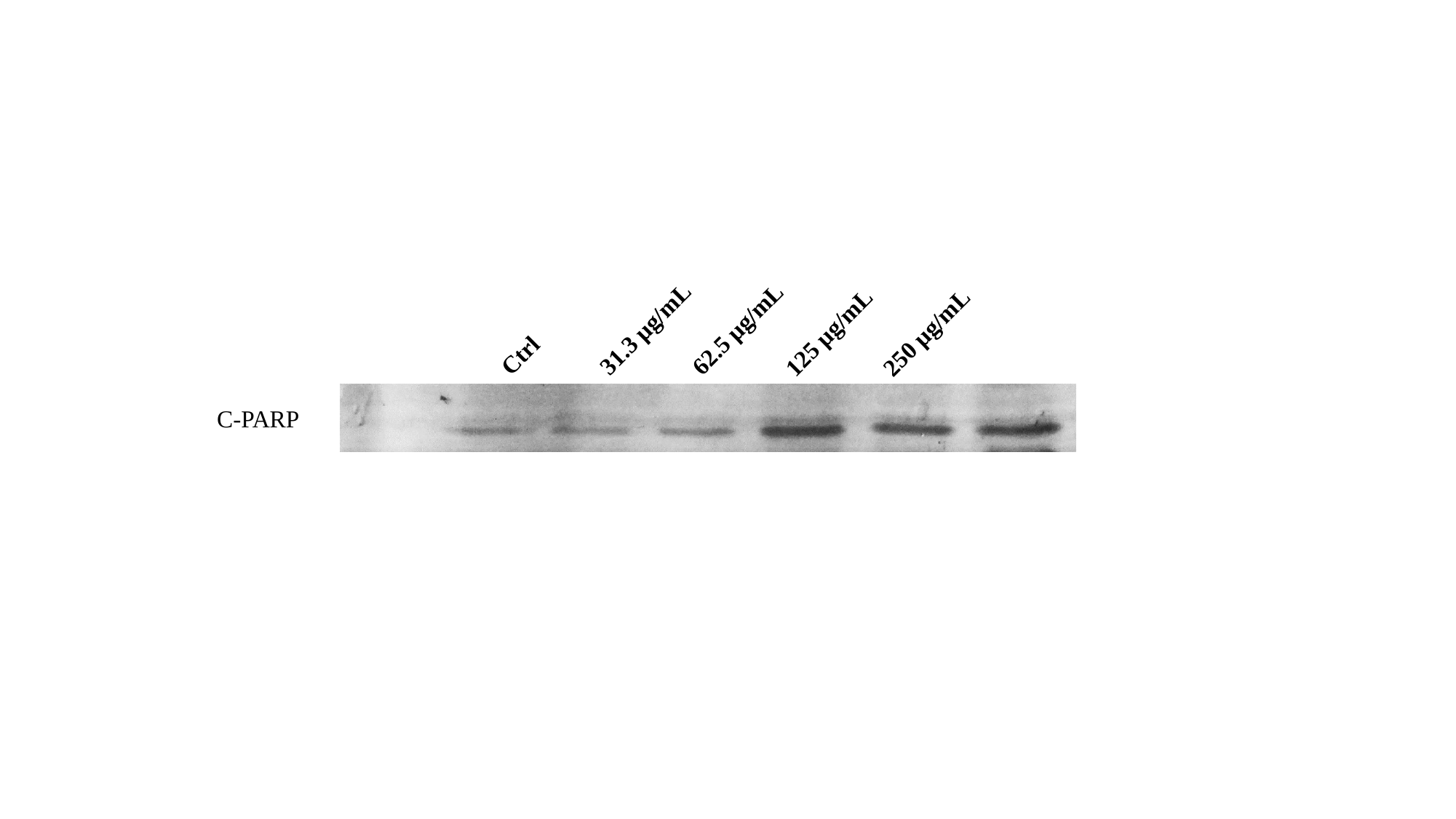

31.3 μg/mL
62.5 μg/mL
125 μg/mL
250 μg/mL
Ctrl
C-PARP

## Slide 9
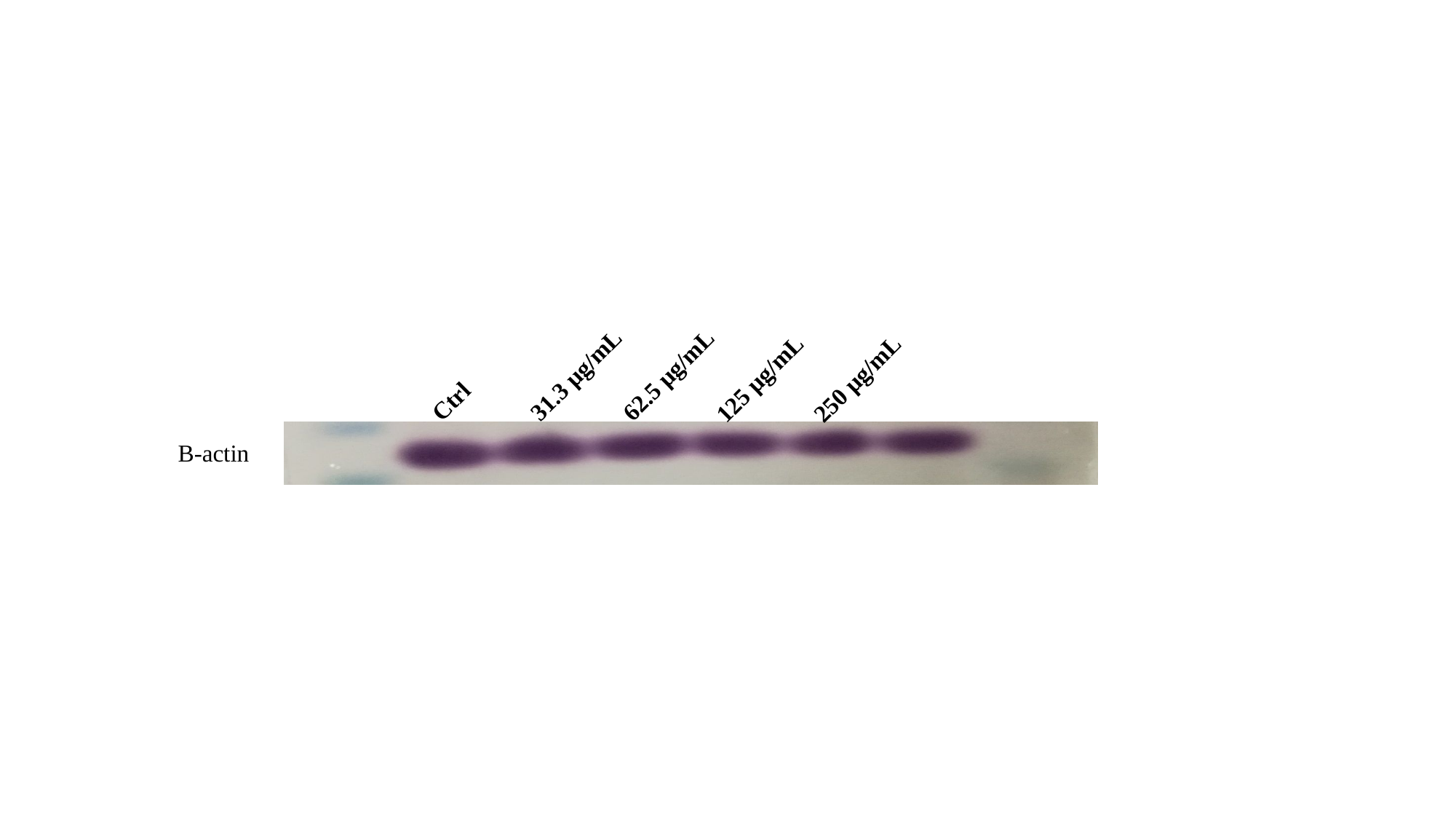

31.3 μg/mL
62.5 μg/mL
125 μg/mL
250 μg/mL
Ctrl
Β-actin
